# The Role of Mitochondrial Enzymes, Succinate-Coupled Signaling Pathways and Mitochondrial Ultrastructure in the Formation of Urgent Adaptation to Acute Hypoxia in the Myocardium

**DOI:** 10.3390/ijms232214248

**Published:** 2022-11-17

**Authors:** Elita Germanova, Natalya Khmil, Lyubov Pavlik, Irina Mikheeva, Galina Mironova, Ludmila Lukyanova

**Affiliations:** 1 Institute of General Pathology and Pathophysiology, 8 Baltijskaya Str., Moscow 125315, Russia; 2 Institute of Theoretical and Experimental Biophysics RAS, 3 Institutskaya Str., Pushchino 142290, Moscow Region, Russia

**Keywords:** urgent adaptation to hypoxia, myocardium mitochondrial enzymes, GPR91, HIF-1α, VEGF, mitochondrial ultrastructure and dynamics, phenotypes with different hypoxia resistance

## Abstract

The effect of a single one-hour exposure to three modes of hypobaric hypoxia (HBH) differed in the content of O_2_ in inhaled air (FiO_2_—14%, 10%, 8%) in the development of mitochondrial-dependent adaptive processes in the myocardium was studied in vivo. The following parameters have been examined: (a) an urgent reaction of catalytic subunits of mitochondrial enzymes (NDUFV2, SDHA, Cyt b, COX2, ATP5A) in the myocardium as an indicator of the state of the respiratory chain electron transport function; (b) an urgent activation of signaling pathways dependent on GPR91, HIF-1α and VEGF, allowing us to assess their role in the formation of urgent mechanisms of adaptation to hypoxia in the myocardium; (c) changes in the ultrastructure of three subpopulations of myocardial mitochondria under these conditions. The studies were conducted on two rat phenotypes: rats with low resistance (LR) and high resistance (HR) to hypoxia. The adaptive and compensatory role of the mitochondrial complex II (MC II) in maintaining the electron transport and energy function of the myocardium in a wide range of reduced O_2_ concentrations in the initial period of hypoxic exposure has been established. The features of urgent reciprocal regulatory interaction of NAD- and FAD-dependent oxidation pathways in myocardial mitochondria under these conditions have been revealed. The data indicating the participation of GPR91, HIF-1a and VEGF in this process have been obtained. The ultrastructure of the mitochondrial subpopulations in the myocardium of LR and HR rats differed in normoxic conditions and reacted differently to hypoxia of varying severity. The parameters studied together are highly informative indicators of the quality of cardiac activity and metabolic biomarkers of urgent adaptation in various hypoxic conditions.

## 1. Introduction

The heart is one of the most intensely working organs, with a very high level of aerobic metabolism. It absorbs about 10% of all oxygen consumed by the organism, though the relative weight of the heart is only about 0.5% of the body weight. Under physiological conditions, the heart workload is to be linearly correlated with oxygen consumption, ensuring conditions of metabolic stability, signal communication and synchronization. Since the heart is a highly energy-consuming organ, there is a strict relationship between oxidative adenosine triphosphate (ATP) synthesis and utilization. Mitochondria are the main source of ATP production necessary for normal cell function and viability. They provide 95% of the ATP generated through oxidative phosphorylation (OXPHOS) for the beating of the heart (6 kg ATP per day) [1,2]. In addition to ATP synthesis, mitochondria regulate a wide range of metabolic processes and signaling pathways in the cell. A crucial role of mitochondria has been established in many aspects of entire cell physiology and pathophysiology in a broad spectrum of diseases, including heart and brain ischemia-reperfusion injury, heart failure, inherited diseases, diabetes, obesity, toxicology, and other pathological conditions [3,4,5,6,7,8,9,10,11,12].

Moreover, the expression of various regulatory protein factors (e.g., GPR91, HIF-1α, VEGF, etc.) and activation of their downstream signaling pathways is also coupled with the activity of mitochondria. These factors and signaling pathways are responsible for many subsequent biological effects, including the development of adaptive processes [10,13,14,15]. Owing to this, mitochondria function as a detector of local stress, including ischemia, hypoxia, toxicity, hyperglycemia, etc.

To date, however, there is no complete understanding of how the myocardial energy apparatus and associated signaling systems work in vivo under various conditions of oxygen deficiency. There is no data on the formation of urgent and delayed compensatory reactions to ensure the functioning of the heart in these conditions. Nevertheless, such data can be used as highly informative indicators of the quality of cardiac activity in various modes of reduced O_2_ concentrations.

It should be taken into account, however, that when there are genetic and phenotypic differences in the resistance of animals to hypoxia, there are animals with high (HR) and low (LR) resistance. These populations, both normoxia and under O_2_ deficit conditions, are characterized by fundamental differences in energy apparatus efficiency, blood oxygen transport function, receptor apparatus, membranes’ condition, neurohumoral regulation, etc. [8,9,10,11,16,17,18]. Differences are also manifested in the contractile function of the myocardium: in HR animals under hypoxia, it is disrupted later and is less pronounced than in LR rats, which correlates with a smaller decrease of the ATP content in the myocardium [8].

There is also no systematic data on the dependence of ultrastructural changes in myocardial mitochondria on the severity and duration of hypoxic effects in vivo. At the same time, it should be borne in mind the complexity of such an analysis in the myocardium due to the features of the mitochondria of cardiomyocytes that distinguish them from most other tissues. They are characterized by three different groups depending on their location and function [19]. These subpopulations of mitochondria not only differ in their morphology and biochemical properties, but can also participate in pathological processes in various different ways, such as ischemic damage, various cardiomyopathies, and have different sensitivity to damage. However, there is no data in the literature on the phenotypic features of the ultrastructure of the mitochondria of the myocardium of LR and HR of animals under hypoxia.

Taking all of this into account, we studied the in vivo simulated effect of a single 1-h exposure of LR and HR rats to three hypoxic regimens (FiO_2_—14%, 10%, 8%). We have researched (a) the urgent reaction of catalytic subunits of mitochondrial myocardial enzymes (NDUFV2, SDHA, Cyt b, COX2, ATP5A-MC I–V) as an indicator of the state of myocardial energy function; (b) the activation of GPR91, HIF-1α and VEGF-signaling pathways in the myocardium, enabling the assessment of their role in the formation of urgent mechanisms of adaptation to hypoxia; (c) changes in the ultrastructure of myocardial mitochondria under these conditions.

Such a comprehensive analysis is necessary to understand the molecular mechanisms of the functioning of the heart in hypoxia. It is also of great diagnostic importance, since in aggregate the results obtained can be used as prognostic criteria for assessing the formation of urgent adaptive myocardial reactions in various conditions of oxygen deficiency.

The obtained results allowed us to establish the special role of MC II in maintaining and preserving the electron transport and energy function of the myocardium during the initial period (the first hour) of hypoxic exposure in a wide range of reduced concentrations of O_2_ (FiO_2_—14–8%).

The results of the work allowed us to establish the special role of MC II in maintaining and preserving the electron transport and energy function of the myocardium during the initial period of hypoxic exposure in a wide range of reduced concentrations of O_2_ (FiO_2_ 14–8%). The features of urgent regulatory interaction of MC II and MC I in myocardial mitochondria at different concentrations of FiO_2_ were revealed. Data on the participation of GPR91, HIF-1α and VEGF in this process were obtained. It has been shown that the ultrastructure of different types of myocardial mitochondria in animals with LR and HR differs in normoxic conditions and reacts differently to hypoxic effects of varying severity.

## 2. Results

### 2.1. Myocardial Mitochondrial Enzymes and Adaptation Factors GPR91, VEGF, HIF-1α under Normoxic Conditions

**Figure 1:**
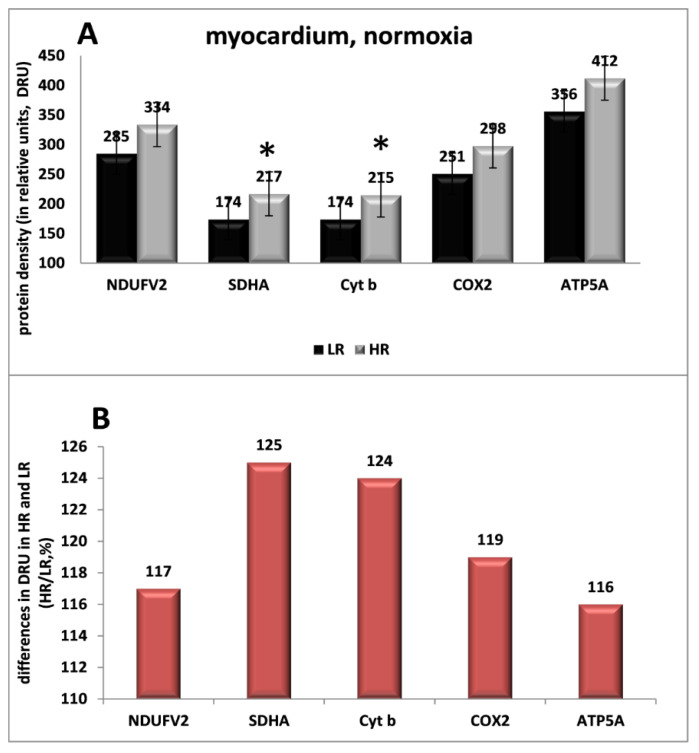
(**A**). The content of the catalytic subunits of mitochondrial complex enzymes MC I–V in the myocardium of HR and LR rats under normoxic conditions (protein density in relative units, DRU): NDUFV2-MC I; SDHA-MC II; Cyt b-MC III; COX2-MC IV; ATP5A-MC V. (**B**). The difference in protein content in the measured subunits in the myocardium of HR and LR rats (HRDRU/LRDRU, %). (*) the differences between the groups are significant, *p* < 0.05.

Our in vivo studies showed that under normoxic conditions (FiO2 = 21%), the content of the catalytic subunits of MC I–V enzymes (NDUFV2, SDHA, Cyt b, COX2, ATP5A) differed in the myocardium of rats with different tolerance to hypoxia (Figure 1). In HR rats, it was 16–25% higher than in LR animals (Figure 1). These data are indicative of phenotypic differences in aerobic energy metabolism in the HR and LR myocardium under normoxy conditions. The power of the respiratory chain in the myocardium of control HR animals is significantly greater compared with LR rats. Moreover, energy metabolism is involved in the formation of individual body resistance to hypoxia.

It was also detected that significant amounts of the succinate-dependent GPR91 receptor, HIF-1α and VEGF signaling protein factors were expressed in the myocardium of both phenotypes under normoxic conditions. The density of GPR91 in the myocardium was the highest compared to other tissues: 1.7 times higher than in the cerebral cortex, 2.9 times higher than in the kidneys, and even more compared to other tissues. As for VEGF, only in striated muscles was its level higher than in the myocardium (2.5 times). In all other tissues, it was significantly lower than in the myocardium: in the cerebral cortex-2 times lower; in the kidneys-4 times lower, etc. Only the level of HIF-1α in the myocardium, cerebral cortex and kidney tissues was approximately the same. The content of these signaling protein factors in the myocardium of HR and LR rats did not differ significantly.

### 2.2. Mitochondrial Ultrastructure in Cardiomyocytes under Normoxic Conditions

Mitochondria of adult cardiomyocytes are relatively static, constrained in their ability to move [20], are in close contact with one another and are divided into three subpopulations (interfibrillar, IFM; subsarcolemmal, SSM; perinuclear, PNM) depending on their location and function [19]. This distinguishes them from the mitochondria of other tissues.

The main portion of mitochondria in cardiomyocytes are the IFM. They are localized in rows between myofibrils and have lamellar cristae involved in the production of ATP for cardiomyocyte contraction and Ca^2+^ signaling. SSM are located under the sarcolemma as separate organelles or small clusters. They feature lamellar cristae used to generate energy for ion channels and transfer intercellular signaling [20,21]. PNM are normally clustered at the poles of the nucleus, are in close contact with the endoplasmic reticulum and are, apparently, involved in transcriptional regulation [20,22]. There are no literature data on the ultrastructural features of mitochondria from HR and LR rat cardiomyocytes.

Our electron-morphometric analysis of the myocardium in HR and LR rats showed that under normoxic conditions each subpopulation of mitochondria had a large number of small organelles (up to 0.25 µm in perimeter). In the PNM subpopulation, it was the largest compared with IFM and SSM: in LR animals, 37% of small mitochondria; in HR rats, 55%. In the IFM subpopulation, they were much less—12% and 15%, respectively; in the SSM subpopulation—15% and 16%. Larger mitochondria were distributed relatively evenly in different subpopulations. However, there were slightly more of them in HR than in LR rats. The exception are the SSM of HR rat myocardium, which were much less than in the IFM and PNM populations (Table 1).

**Figure 2:**
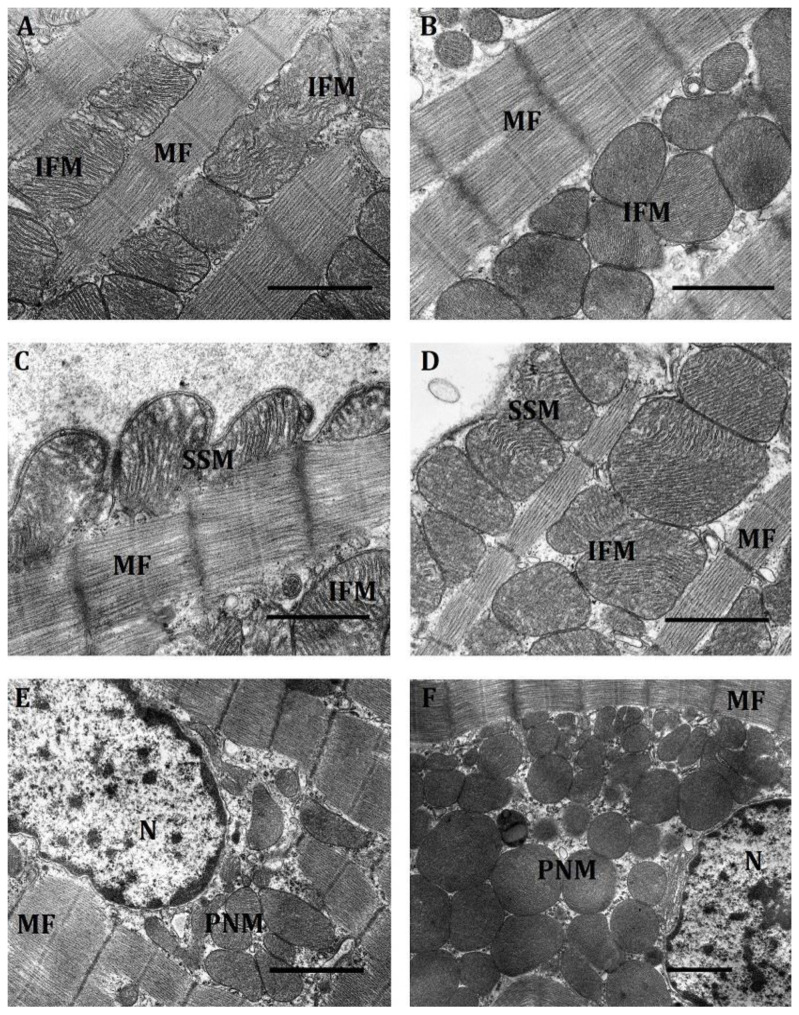
The ultrastructure of a part of a cardiomyocyte in rats with low (**A**,**C**,**E**) and high (**B**,**D**,**F**) resistance to oxygen deficit under normoxic conditions. Representative electron microscopy images showing intermyofibrillar (**A**,**B**), subsarcolemmal (**C**,**D**) and perinuclear (**E**,**F**) mitochondria; IFM, SSM and PNM are interfibrillar, subsarcolemmal and perinuclear mitochondria, respectively; MF, myofibrils; N, nucleus. Scale bar, 0.5 μm.

Under normoxic conditions, all three subpopulations of mitochondria in LR and HR rats had initial structural differences. Thus, IFM in both phenotypes clearly resided between rows of myofibrils. However, in LR rats they were normally located between myofibrils in one row; in HR animals, in two or more rows (Figure 2A,B).

The IFM subpopulation of HR rats featured an irregularly rounded shape, organelles closely packed to one another, pronounced intermitochondrial contacts, an electron-dense matrix and densely packed cristae, which, as a rule, had a transverse orientation.

In contrast, IFM of LR rats were of a more irregular oval-elongated shape. Intermitochondrial contacts were less pronounced or were absent altogether. Cristae were arranged more loosely and were clearly separated by an intercristal space. They are distinguished by a less pronounced orientation, which can be either horizontal, vertical or mixed (Figure 2A). All of this indicates that, as compared with HR, myocardial LR rat mitochondria are in a less energized state.

There were also fundamental differences in the location of the SSM subpopulation in the myocardium of HR and LR rats. The location of organelles in evaginations of the sarcolemma was characteristic of LR rat myocardium; this lent to its tortuous shape (Figure 2C). In the HR rat myocardium, SSM were less submerged under the sarcolemma, which was therefore less tortuous (Figure 2D). Unlike IFM, SSM cristae had a lamellar structure. It was especially well pronounced in LR animals. Cristae, as a rule, were rather ordered, had a longitudinal arrangement and were separated by wide intercristal spaces. As a result, the mitochondrial matrix of SSM in LR rats was of average electron density. SSM of LR rats were distinguished by a more ordered arrangement of cristae and a more electron-dense matrix. The average number of SSM in the two groups of animals did not differ significantly (Table 1). It was 9–11 units/10 µm^2^. The proportion of small mitochondria among the considered organelles in both types of animals averaged 18% (LR rats) and 16% (HR rats) of the total.

Under normoxic conditions, the PNM population comprised the largest number of small mitochondria that in HR rats were 55% and in LR animals, 35% of the total number of mitochondria, which sharply distinguished them from IFM and SSM (Table 1). Herewith, PNM of HR rats were 1.7 times larger than those of LR animals, and their number was 1.6 times greater. Accordingly, the perinuclear region normally localized at the poles of the nucleus was also much larger in HR rats (Figure 2F). In the latter, most PNM contained in it were of a round shape, had a dense matrix and regularly organized cristae characteristic of this type of organelles, were more motile and not fixed by myofibrils. Still, irregular-shape fused mitochondria also occurred (Figure 2E,F). Most PNM in HR rats communicate with one another, forming contacts of the type of “kissing junctions” between adjacent mitochondria [23] and thus creating clusters.

In contrast, in LR rats PNM were much more small-numbered and more polymorphic. Oval, elongated, tortuous shapes were observed (Figure 2E,F). They were located more diffusely, though there were also associated groups making contact by the type of “kissing junctions” [23] or connected by nanotunnels. Their matrix was dense, but more clarified than in HR rats. PNM in both phenotypes of animals were located at the poles of the nucleus, which was localized, as a rule, in the middle of the cell (Figure 2E,F).

### 2.3. Urgent Reaction of Mitochondrial Enzyme Subunits of the Substrate Site of the Myocardial Respiratory Chain (SDHA, NDUFV2) to a Single Hypoxic Exposure In Vivo

The urgent dynamics of SDHA subunit expression in the myocardium in all modes of single hypoxia (HBH, FiO2 = 14–8%) was of a two-phase nature. In the first 45–60 min of hypoxic exposure, an urgent induction of SDHA expression was observed, followed by a decrease in its intensity. It started at the end of hypoxic exposure and ended in the post-hypoxic period. The dynamics of this subunit expression differed in LR and HR animals.

**Figure 3:**
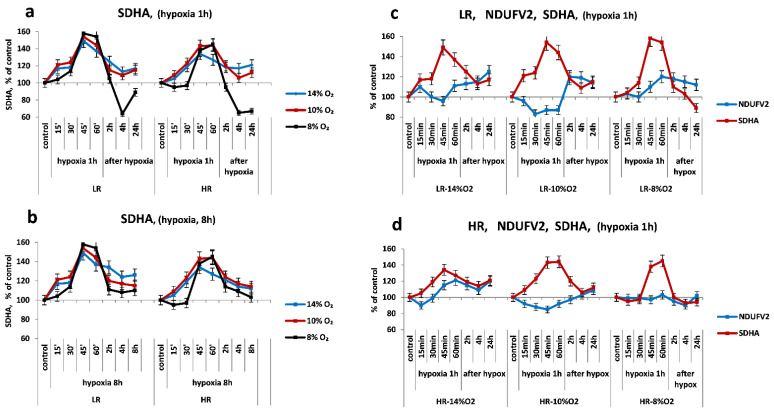
Dynamics of the expression of catalytic subunits of respiratory enzymes (NDUFV2, SDHA-MC I-MC II) in LR and HR rat myocardium during a single 1-h hypoxic exposure (min) and in the post-hypoxic period (2 h and 24 h). Leftward: Effect of 1-h (**a**), and 8-h (**b**) hypoxic exposure (FiO_2_–14%, 10%, 8%) on the dynamics of SDHA expression in the myocardium of LR and HR rats. On the right: Effect of 1-h hypoxic exposure (FiO_2_–14%, 10%, 8%) on the dynamics of SDHA and NDUFV2 expression in the myocardium of LR (**c**) and HR (**d**) rats. The ordinate axis shows Western blot analysis data in % of the control taken as 100%. Along the abscissa axis is the time of tissue sampling during hypoxia (minutes), and in the post-hypoxic period (hours).

In mild to moderate hypoxia, the expression of the SDHA subunit began to increase after 15 min and reached maximum values after 45–60 min (150–160% in LR animals and 135–145% in HR animals). At the end of the 1-h hypoxic exposure or immediately after its termination, the SDHA level began to decrease. In the post-hypoxic period after 2–4 h, it decreased, respectively, to 115–120% and stabilized at these values over the next 24 h (Figure 3a). In severe hypoxia (FiO2 = 8%), the urgent expression of SDHA grew more slowly, especially in HR rats. Nevertheless, after 45–60 min of hypoxic exposure, the enzyme level reached the same values as in more weakened hypoxic regimes. In the post-hypoxic period, the SDHA level returned to its initial values after 2 h and even decreased slightly relative to the control by 4 h in order to remain at this level for the next 24 h (Figure 3a).

The intensity of urgent SDHA expression did not depend on the duration of a single hypoxic exposure. With all hypoxic modes used, an increase in the duration of hypoxic exposure to 4–8 h did not significantly affect the dynamics of SDHA expression. The SDHA level always began to decrease after 45–60 min, even if the hypoxic effect continued. However, it was slower than during the reperfusion period. Even after 8 h of hypoxic exposure, the amount of the subunit exceeded the initial level (Figure 3b).

Thus, in the mitochondria of the myocardium—the main substrate of oxidation for which, in normoxic conditions, are fatty acids—there is an obligate increase in SDHA expression in the first 15–30 min of hypoxic exposure of any severity, which reflects a relatively short-term (about an hour) activation of succinate oxidation (MC II). In all hypoxic regimes, it did not depend on the duration of hypoxic exposure. Consequently, the urgent activation of succinate oxidation in response to any hypoxic effect is a self-regulating compensatory-adaptive process. During severe hypoxia, this process developed in the myocardium of HR rats more slowly than in LR (after a 30-min latent period).

The urgent dynamics of the NDUFV2 subunit under acute hypoxic exposure was also two-phased and was always combined with the dynamics of the SDHA subunit, although it was fundamentally different (Figure 3c,d).

In mild hypoxia (FiO_2_ = 14%), the level of NDUFV2 in the myocardium of HR and LR rats in the first 30–45 min varied, not significantly exceeding the initial values. However, at the very end of the 1-h hypoxic exposure, it began to increase against the background of either a maximum increase in SDHA expression or its incipient decrease. NDUFV2 expression continued to increase during the post-hypoxic period for 24 h.

On the contrary, after 15 min of hypoxic exposure at FiO_2_ = 10%, a sharp decrease in the level of NDUFV2 was observed in the myocardium of HR and LR compared to the control and in contrast to the increase in SDHA expression. However, immediately after hypoxic exposure, the level of the subunit was restored, and over the next 24 h it significantly exceeded the initial values (Figure 3c,d).

At FiO_2_ = 8%, the level of NDUFV2 in the HR myocardium did not change significantly throughout the hypoxic exposure and in the post-hypoxic period (Figure 3d). However, in the LR myocardium, NDUFV2 expression began after a 30-min latency period and persisted after hypoxia (Figure 3c).

**Figure 4:**
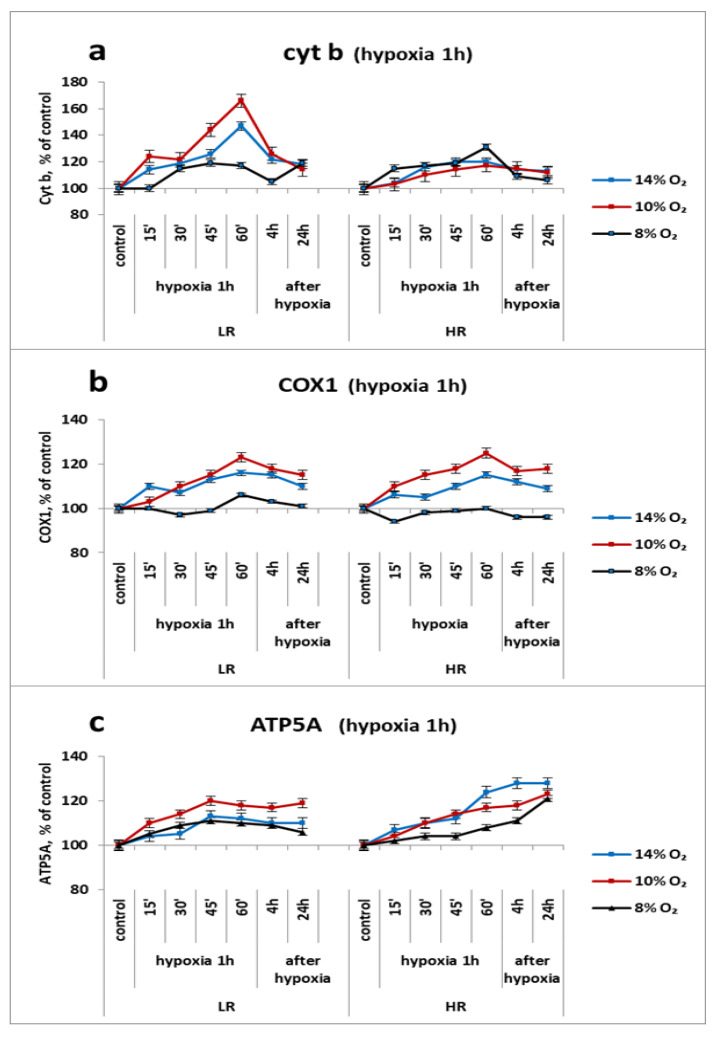
Dynamics of the expression of catalytic subunits of respiratory enzymes (Cyt b (**a**), COX2 (**b**) and ATP5A (**c**) in LR and HR rat myocardium during a single 1-h hypoxic exposure (HBH, FiO_2_–14, 10, 8% O_2_) and in the post-hypoxic period (see Figure 2).

As in the case of SDHA, an increase of the duration of single hypoxic exposure to 8 h did not fundamentally affect the dynamics of the process (the data are similar to Figure 3b and not shown). This is also indicated by an increase in the expression of the COX2 subunit of cytochrome oxidase (COX), an enzyme of the terminal site of the respiratory chain (MC IV), in both animal phenotypes; the increase was observed throughout the entire period of hypoxic exposure at FiO_2_ of 14% and 10% (Figure 4b). The level of this enzyme slightly decreased at severe hypoxia (FiO_2_ = 8%) but remained within the initial values. It was also preserved in the post-hypoxic period (Figure 4b). Thus, the 1-h exposure to HBH of different severity (FiO_2_ = 14–8%) did not disturb the function of the cytochrome oxidase.

The expression of the ATP5A subunit during hypoxic exposures of any severity was also increased. In the post-hypoxic period, although it decreased slightly, its level remained above the baseline values.

Thus, the data obtained allow us to draw the following conclusions:

(1) Myocardium mitochondrial enzymes finely differentiate the decrease in the oxygen content in the environment and perform the function of a regulatory signaling mechanism that activates urgent adaptive reactions. (2) In the myocardium, any hypoxic regimen used by us is accompanied by an urgent activation of succinate oxidation, which leads to the expression of the cytochrome site enzymes of the respiratory chain and ATP synthase and activation of energy synthesis. (3) The process of switching over the respiratory-chain substrate flows to succinate oxidation in the early period of hypoxic exposures is an urgent adaptive compensatory reaction of energy metabolism, which ensures the stabilization of the myocardium function under these conditions.

### 2.4. Urgent Reaction of Mitochondrial Enzymes’ Subunits of the Respiratory Chain Cytochrome Site in the Myocardium (Cyt b, COX2 and ATP5A) to a Single Application of Hypoxia In Vivo

There is an opinion based on the results of experiments conducted under in vitro conditions at very low values of pO_2_ (1–5% O_2_) that the use of succinate as an oxidation substrate leads to a decrease in respiration, and that the activation of succinate oxidation cannot ensure the full-fledged energy status of the cell. In our experiments, however, in hypoxic regimens of mild and moderate severity (FiO_2_ = 14–10%) we observed the activation of not only enzymes of the cytochrome site of the myocardial respiratory chain (Cyt b, MC III; COX2, MC IV) but also of ATP5A (ATP synthase, MC V) that occurred during the period of increased MC II activity. Under conditions of severe hypoxia (FiO_2_ = 8%), the activity of these enzymes decreased, but remained above the baseline values (Figure 4a–c).

Thus, already at 15 min of the first 1-h hypoxic exposure, both phenotypes showed an increase in Cyt b subunit expression that reached maximum values after 1 h and correlated with the dynamics of SDHA expression. After the end of the exposure, the level of the subunit gradually decreased, but even after 24 h it exceeded the control values. The intensity of the reaction was more pronounced in LR rats and depended on the severity of hypoxia. At FiO_2_ of 14% and 10%, the level of Cyt b in LR animals increased to 140% and 160%, respectively, and at FiO_2_ of 8% it decreased to 120% (Figure 4a). In HR rats under all hypoxic regimens, it did not exceed 110% (Figure 4a). An increase in the level of the Cyt b (MC III) subunit can be indicative of an increase in the electron transport function on this segment.

This is also indicated by an increase in the expression of the COX subunit of cytochrome oxidase (COX), an enzyme of the terminal site of the respiratory chain (MC IV), in both animal phenotypes; the increase was observed throughout the entire period of hypoxic exposure at FiO_2_ of 14% and 10% (Figure 4b). The level of this enzyme slightly decreased at severe hypoxia (FiO_2_ = 8%) but remained within the limits of the baseline values. It was also preserved in the post-hypoxic period (Figure 4b). Thus, a 1-h exposure to different-severity HBH (FiO_2_ = 14–8%) did not disturb the function of the cytochrome oxidase.

The expression of the ATP5A subunit during hypoxic exposures of any severity was also increased. In the post-hypoxic period, though it decreased slightly, its level remained above the baseline values.

Thus, the data obtained allow us to draw the following conclusions:

(1) Myocardium mitochondrial enzymes finely differentiate the decrease in the oxygen content in the environment and perform the function of a regulatory signaling mechanism that activates urgent adaptive reactions. (2) In the myocardium, any hypoxic regimen used by us is accompanied by an urgent activation of succinate oxidation, which leads to the expression of the cytochrome site enzymes of the respiratory chain and ATP synthase and activation of energy synthesis. (3) The process of switching over the respiratory-chain substrate flows to succinate oxidation in the early period of hypoxic exposures is an urgent adaptive compensatory reaction of energy metabolism, which ensures the stabilization of the myocardium function under these conditions.

### 2.5. Urgent Expression of Succinate-Dependent Adaptation Factors (GPR91, VEGF, HIF1-α) in the Myocardium with a Single Hypoxic Exposure of Varying Severity

The ability of the myocardium during acute hypoxia to mobilize not only the regulatory centers of the respiratory and cardiovascular systems, but also the mechanisms associated with production of the energy and ensuring stabilization of the heart for a certain period of time is hardly possible without the involvement of associated signaling systems of urgent adaptation.

Taking into account the revealed special compensatory role of succinate oxidation during this period, we investigated the participation in this process of succinate-dependent adaptive protein factors GPR91, VEGF and HIF1-α.

***Hypoxia andHIF-1a expression in the myocardium.*** According to modern concepts, the leading role in the formation of adaptation to hypoxia belongs to a specific protein transcription factor induced by hypoxia (hypoxia inducible factor, HIF-1), which functions as a transcription activator and a key regulator of various cellular and systemic reactions to hypoxia. Hypoxic stabilization of one of its main subunits, HIF-1α, depends on succinate. Succinate inhibits prolyl hydroxylase reactions responsible for the ubiquitin-dependent proteolysis of HIF-1α, and thereby contributes to its accumulation [24,25,26,27].

In this regard, revealed activation of succinate oxidation in the myocardium in response to a single application of hypoxia also implies activation of HIF-1α expression. Nevertheless, the regulatory relationship between the content of HIF-1α in the myocardium, the state of the respiratory chain and the role of succinate in this process during hypoxia still remains open today. It should be borne in mind that there is no correlation in the heart between the initial content of succinate and HIF-1α. The initial level of HIF-1α in the myocardium is very high, while the succinate content is almost the lowest of all tissues [28,29].

**Figure 5:**
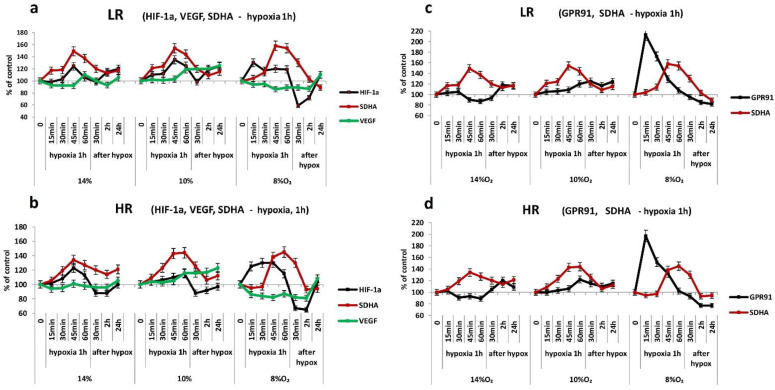
Dynamics of the expression of HIF1-α, VEGF, SDHA (**a**,**b**), GPR91 and SDHA (**c**,**d**) in LR and HR rat myocardium during a single 1-h hypoxic exposure (HBH, FiO_2_—14, 10, 8% O_2_) and in the post-hypoxic period (For the rest, see Figure 2).

According to our data, in the myocardium of both phenotypes, the dynamics of urgent expression of HIF1-α in response to 1-h exposure to mild and moderate hypoxia (FiO_2_ = 14–10%) correlated with the dynamics of SDHA expression, i.e., with the activation period of succinate oxidation (Figure 5a,b). However, an increase in the level of HIF1-α in both cases was observed after a significant latent period, against the background of already high level of SDHA. The decrease in HIF1-α expression at first also occurred in parallel with the decrease in SDHA levels, but 30 min after the end of hypoxic exposure, a secondary increase in HIF1-α levels was observed, despite the stabilization of SDHA values.

On the contrary, in severe hypoxia (FiO_2_ = 8%), the induction of urgent HIF1-α expression preceded the increase in SDHA levels. Already after 15 min of hypoxic exposure, the increase in HIF1-α levels in the myocardium of HR and LR rats was 120–125% and was stably maintained at this higher level for 1 h (Figure 5a,b). SDHA expression began to increase only after 30 min of hypoxia. Immediately after the end of hypoxic exposure, the expression of HIF1-α decreased in parallel with the decrease in SDHA levels, but increased again after 30 min of the post-hypoxic reoxygenation period, regardless of the SDHA values (Figure 5a,b).

Thus, any applied regime of acute 1-h hypoxic exposure stimulated the urgent expression of HIF1-α in the myocardium of both animal phenotypes. However, depending on the severity of hypoxic exposure, this process was controlled by various mechanisms. During the action of mild and moderate hypoxia, the dynamics of HIF1-α was succinate-dependent. This dependence was absent in the early post-hypoxic period and in the next 24 h. In severe hypoxia, the urgent induction of HIF1-α expression significantly outpaced the activation of succinate oxidation. The subsequent decrease in its expression in the first hours of reoxygenation again correlated with a decrease in SDHA levels, but was replaced at a later period (after 24 h) by its reciprocal increase. In all cases, changes in the content of HIF1-α in the myocardium of HR rats were significantly less pronounced than in LR animals.

***Hypoxia and VEGF expression in the myocardium***. HIF-1a is known to increase the expression of ***VEGF***, which is associated with neovascularization in hypoxic-ischemic tissues under hypoxic conditions [30,31].

In our experiments, however, there was no correlation between the dynamics of HIF-1α and VEGF in the myocardium during the acute period of a 1-h hypoxic exposure to any regimen in both animal phenotypes (Figure 5a,b). Thus, despite the bell-shaped dynamics of HIF-1α and SDHA expression characteristic of mild hypoxia, the VEGF level under these conditions did not significantly differ from the baseline level. In the post-hypoxic period, it did not change, either. At moderate hypoxia, VEGF expression increased only at the very end of exposure, against the background of the incipient decrease in HIF-1α and SDHA expression, and was maintained at this level during the post-hypoxic period for 24 h.

In severe hypoxia, we observed a tendency to decrease VEGF expression, despite the complex dynamics of HIF-1α (primary increase in its expression independent of succinate, followed by its sharp post-hypoxic inhibition for 2 h). The return of the VEGF level to the baseline values and even a slight excess of them, correlating with the restoration of the HIF-1a content, was observed only after 24 h (Figure 5a,b). In all hypoxic regimes, the dynamics of VEGF expression in the HR and LR myocardium did not differ (Figure 5a, b).

Thus, urgent expression of VEGF in the myocardium was not induced with a single 1-h hypoxic exposure in a wide range of reduced O_2_ concentrations (FiO_2_ = 14–8%). Consequently, VEGF is not involved in the formation of urgent compensatory adaptive reactions in the myocardium.

***Hypoxia and expression of GPR91 in the myocardium.*** GPR-coupled receptor GPR91 acts as a sensor of extracellular succinate, which is its ligand. It has been found in most aerobic tissues, but its baseline content in the myocardium is significantly greater than in other tissues. [32]. Similarly the myocardium is characterized by the highest activity of SDHA compared to other tissues, but a very low content of endogenous succinate [32]. In the heart, GPR91 is localized mainly in the sarcolemmal membrane and T tubules [33,34]. Signaling GPR91-dependent intracellular pathways include activation of PI3K, ERK1/2, JNK, p38 MAPK, COX-2 and PGE2, which mediate many subsequent biological effects [35,36,37].

The dependence of the receptor activity on succinate suggests that under hypoxic conditions, switching of the myocardial respiratory chain to succinate oxidation may correlate with an increase in GPR91 expression. This is exactly the reaction we observed in the cerebral cortex in response to a single hypoxic exposure of various modes [9,10].

In the myocardium, however, there was no such correlation. According to our data, in the myocardium of both rat phenotypes, there were no significant changes in the level of GPR91 during 1-h exposure to mild and moderate hypoxia (FiO_2_ = 14–10%), despite an increase in SDHA expression. A slight increase in the receptor was observed only in the post-hypoxic period (Figure 5c,d).

In severe hypoxia (FiO_2_ = 8%), however, the activation of urgent expression of GPR91 in the myocardium of HR and LR rats significantly outpaced the induction of SDHA. After 15 min, the GPR91 level reached its maximum values (220%), and then it sharply decreased, despite the continued hypoxia impact and a gradually increasing SDHA level. This effect may reflect internalization and then desensitization of the receptor [36,37].

Thus, in the myocardium, single hypoxic exposures of mild and moderate severity were accompanied by the expression of only HIF-1a. In contrast, in severe hypoxia, induction of both HIF-1a and GPR91 expression was observed, significantly outpacing SDHA expression. This may be due to the need in these conditions to mobilize a wider range of basic adaptive signaling systems that could ensure the preservation of myocardial contractile function.

### 2.6. Ultrastructure of Three Subpopulations (IFM, SSM, PNM) in the Myocardium of LR and HR Rats after a Single 30-min Different-Severity Hypoxic Exposure

a. - Effect of hypoxia on the ultrastructure of IFM in the myocardium of HR and LR rats

**Figure 6:**
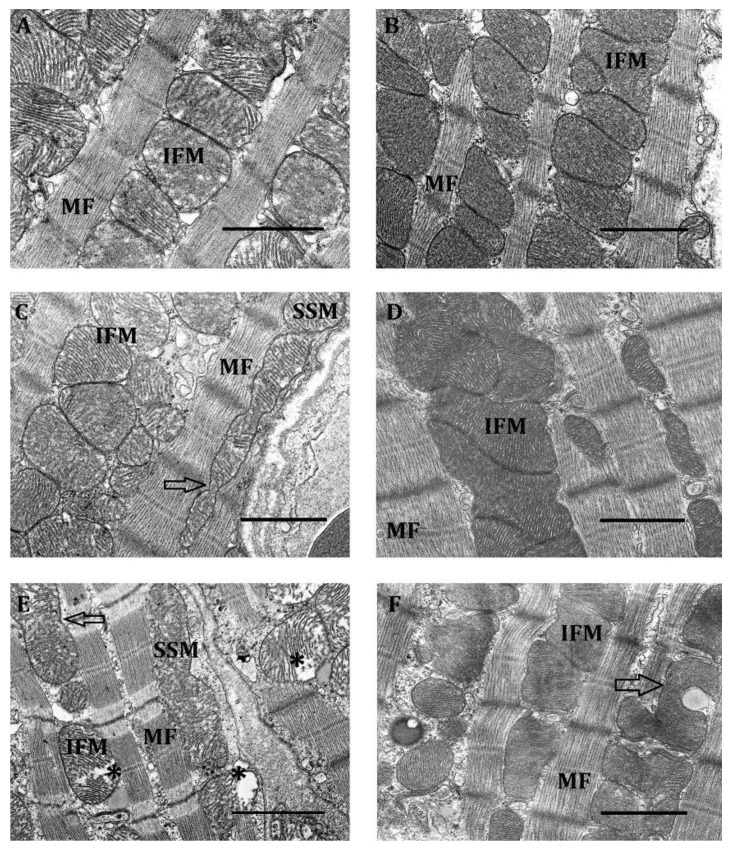
The ultrastructure of the intermyofibrillar region of a cardiomyocyte in rats with low (**A**,**C**,**E**) and high (**B**,**D**,**F**) resistance to oxygen deficit in mild (FiO_2_ = 14%, (**A**,**B**)), moderate (FiO_2_ = 10%, (**C**,**D**)) and severe (FiO_2_ = 8%, (**E**,F)) hypoxia. Tissue samples were taken 30 min after the start of the respective hypoxic training. IFM, interfibrillar mitochondria; MF, myofibrils. Arrow—elongated interlocked mitochondria; asterics—lysed mitochondria. Scale bar, 0.5 μm.

Under the action of mild hypoxia (FiO_2_ = 14%), most IFM in HR and LR rats preserved a rounded or slightly oval shape. However, the average values of area of the organelles decreased, which is indicative of their compaction, especially in HR rats, since the average values of their perimeters also decreased (Table 2). In both cases, the density of contacts between mitochondria was found to increase (Figure 6A,B). At the same time, in the LR group the arrangement of cristae was observed to become chaotic and disorganized, and their order was disturbed (Figure 6A).

Another feature of this hypoxic effect on the myocardium was an increase in the number of small IFM (of up to 0.25 µm in perimeter) per unit area. In LR rats, it increased three-fold as compared to the control; in HR animals, 1.5-fold (Table 3).

Thus, it follows from the results obtained that a mild hypoxic exposure (FiO_2_ = 14%), considered to be subthreshold when used in vivo, leads after 30 min to minor ultrastructural changes in IFM, accompanied by an increase in the condensation of mitochondria, i.e., an increase in the degree of their energization.

Moderate hypoxia (FiO_2_ = 10%) led to more significant changes in the IFM structure of both types of animals. Thus, the average area of the organelles increased as compared with the control group and with the previous hypoxic regimen (Table 2). Herewith, the size of IFM also slightly increased.

We observed no significant changes in the total number of IFM in LR rats with hypoxia of moderate severity compared with the control, although there was a tendency of its decrease compared with mild hypoxic exposure (Table 3). At the same time, the number of small mitochondria in this subpopulation in LR rats increased, but less than at mild hypoxia (1.8 times). In contrast, in HR animals the total number of IFM decreased significantly compared with the control and with the previous hypoxic regimen (by 56% and 35%, respectively), as did the number of small organelles (Table 3).

In both phenotypes, this hypoxic regimen contributed to combining IFM into large associates (Figure 6C,D). Mitochondria in such associates closely adhered to one another, forming intermitochondrial contacts (Figure 6C,D).

In HR rats the shape of IFM in associates was rather heterogeneous. The matrix of such mitochondria was denser than in the control and in the previous hypoxic regimen. Cristae were clearly organized and had minimal intercristal distances. Herewith, their lamellar or laminar structure, as well as their transverse orientation, were viewable better than in the control. Thus, IFM combined into associates were highly energized, which is consistent with the literature data [38,39]. However, a small number of IFM in the HR group preserved a linear single-row arrangement. Mitochondria were connected by spherical intermitochondrial bridges with a vacuole inside [38] and formed a chain. These intercalated spherical bridges were located at the level of the Z-lines of myofibrils (Figure 6D).

Transition from linearly arranged IFM to associates was also observed in the myocardium of HR rats. In this case, the organelles were of a predominantly rounded and oval-rounded shape. Intermembrane contacts were preserved but were less dense than in HR rats. The density and order of LR rat IFM cristae under this hypoxic regimen also increased compared to the previous regimen. Herewith, the lamellar (laminar) structure of cristae and their transverse orientation were clearly visible. However, in some IFM a chaotic arrangement of cristae and their partial lysis persisted (Figure 6C).

Proceeding from canonical views, the trend of IFM ultrastructure changes at a mild hypoxic regimen indicated that this subpopulation of mitochondria was in a highly energized state. This was especially pronounced in the myocardium of HR rats.

Severe hypoxia (FiO_2_ = 8%) led to more pronounced changes in IFM ultrastructure compared with two previous hypoxic regimens. Thus, in the myocardium of LR rats, the number of small IFM sharply rose again (2.8-fold), while in HR animals it increased only 1.4-fold. The average IFM area of LR rats decreased by 23% compared to the control (Table 2). In contrast, in HR rats these parameters remained at the control level. In both phenotypes, the number of small IFM increased sharply again: in LR rats, 2.8-fold; in HR rats, 1.4-fold. The number of IFM in severe hypoxia in LR rats increased compared to the control due to small mitochondria (Table 3); in HR rats, quite the opposite, it decreased.

Under conditions of severe hypoxia, signs of insufficiency of the cardiomyocytes’ contractile apparatus appeared in the myocardium of both phenotypes. In HR rats, they were expressed in “melting”, a diffuse lysis of myofibrils, violation of their normal orientation near the intercalated discs (emergence of a curved shape) (Figure 6E,F). In LR rats, contraction damage manifested itself in clearcut Z-lines and focal lysis of MF near the intercalated discs (Figure 6E). In both types of animals, local cytosol clarifications (myocytolysis) appeared between MF [40,41]. Considering that the main function of IFM is to produce ATP [1,41,42], the described changes of MF ultrastructure may reflect a decrease in the energy function of this mitochondrial subpopulation.

The dissociation of IFM associates observed in the myocardium of both phenotypes may also be related to this. Most of the IFM after this dissociation had an irregular shape. Herewith, HR animals retained only single paired IFM with not very tight fit one to another, which, as is known, is consistent with a decrease in respiration [19]. The number of intermitochondrial contacts was smaller. Large slit-like spaces containing vacuoles appeared between the organelles (Figure 6F). Part of the IFM were at a large distance from one another. Local clarifications of cytosol were seen between them. The IFM matrix of HR rats, though remaining electron-dense, became slightly clarified. In some IFM, cristae were pronounced and maintained a horizontal orientation. However, there were organelles with fuzzy and disordered cristae (Figure 6F).

In LR rats, changes of IFM ultrastructure were even more pronounced. The total number of mitochondria in this subpopulation sharply increased due to small organelles (2.8 times). There were no associated IFM. Only single mitochondria that were apart from one another were seen. Their matrix was very clarified due to increased intercristal distances. A significant part of cristae was lyzed (Figure 6E).

Thus, a 30-min severe hypoxic exposure (FiO_2_ = 8%) led to serious violations of the IFM ultrastructure characteristic of various myocardial pathologies, which was indicative of a decrease in the efficiency of the energy apparatus.

The obtained data on the effect of different acute hypoxia regimens on myocardial IFM indicate the ability of this subpopulation of mitochondria in vivo to subtly distinguish between gradual changes in the content of O_2_ in the medium. Herewith, a differentiated energy-dependent modification of the IFM ultrastructure occurs in the FiO_2_ range of 14–10%, which ensures myocardial operability under conditions of moderate oxygen deficit. As our research shows, optimal conditions for such an ultrastructural rearrangement are created at FiO_2_ of 10%. In contrast, the ultrastructural pattern of IFM at FiO_2_ of 8% is characterized by evident signs of de-energization, despite the absence of violations in the state of respiratory-chain cytochrome segment enzymes.

b. Effect of hypoxia on the ultrastructure of SSM in the myocardium of LR and HR rats

SSM represent a small peripheral subpopulation located the closest to the sarcolemmal membrane. Because of this, they are better supplied with oxygen and, apparently, experience its deficit less [7].

At mild hypoxia, the average area and perimeter of SSM in HR and LR rats remained close to the control values (Table 2), though their total number and the number of small mitochondria decreased in both cases (Table 4).

**Figure 7:**
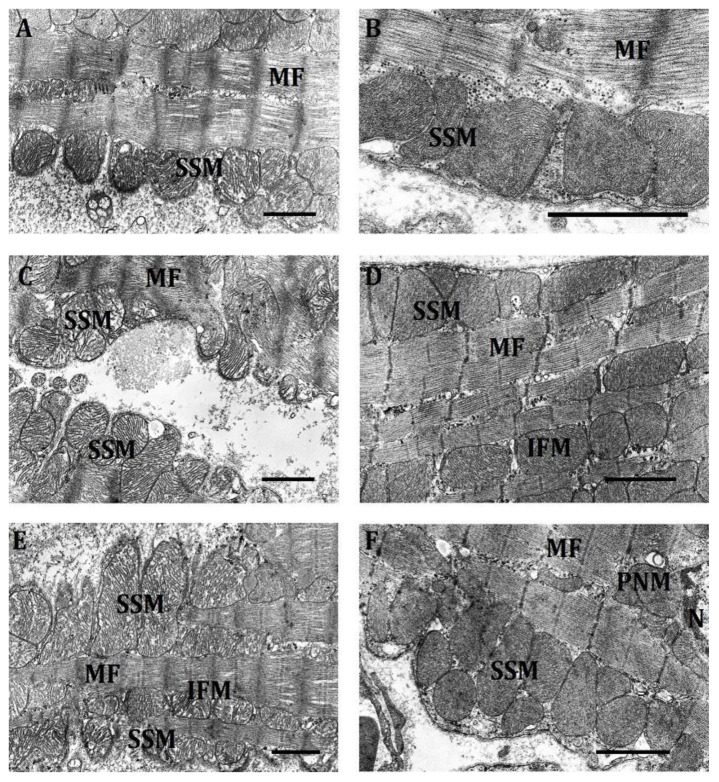
The ultrastructure of a subsarcolemmal region of a cardiomyocyte in rats with low (**A**,**C**,**E**) and high (**B**,**D**,**F**) resistance to oxygen deficit in mild (FiO_2_ = 14%, (**A**,**B**)), moderate (FiO_2_ = 10%, (**C**,**D**)) and severe (FiO_2_ = 8%, (**E**,**F**)) hypoxia. Tissue samples were taken 30 min after the start of the respective hypoxic training. SSM, subsarcolemmal mitochondria; MF, myofibrils. Scale bar, 0.5 μm.

We found no significant changes in the localization of SSM in either HR or LR rats. SSM of LR rats were characteristically located in invaginations of the sarcolemmal membrane, the length of which, as a rule, was equal to one sarcomere. This localization lent the membrane a tortuous shape not observed in SSM of HR animals (Figure 7A). The shape and structure of SSM slightly changed. Prevailing in LR rats were round-shape SSM, though in the control they were characterized by an oval shape. Cristae became more ordered, and the matrix got denser. Along with this, SSM with lyzed cristae emerged in places. In HR rats, irregular-shape SSM with an electron-dark matrix and ordered cristae dominated. There was no tight fit of mitochondria to one another in both phenotypes. Thus, at mild hypoxic exposure we observed only unreliable and difficult-to-interpret changes of SSM ultrastructure. Nevertheless, they suggest the tendency to an increase of SSM condensation and energization.

At hypoxia of moderate severity, the character of SSM localization was preserved; in LR rats, unlike HR, the organelles were located mainly one each in invaginations of the sarcolemmal membrane. As for the area of SSM, it increased in both phenotypes; their total number and the number of small mitochondria continued to decrease (Table 2 and Table 4). All this indicates an increase in the size of SSM under this hypoxic effect, especially pronounced in LR rats, which may indicate activation of cell fusion processes. Indeed, LR animals did sporadically feature septate mitochondria, as well as chains of several fused organelles with constrictions that occupied a distance of several sarcomeres (Figure 6C). Herewith, clearly pronounced was the tightening of contacts between SSM, as well as an increase in the density and order of cristae, their laminar structure and transverse or longitudinal orientation. However, this group also comprised mitochondria with chaotic arrangement of cristae and even with their partial lysis.

In the SSM subpopulation of HR rats, indications of mitochondrial fusion were less pronounced. However, there was a clear tendency towards the formation of mitochondrial associates (Figure 7D). Mitochondria very closely adhered to one another, forming intermitochondrial contacts (Figure 7D). Some SSM of HR rats were of irregular shape, but mostly organelles retained an ordered arrangement of cristae and an electron-dense matrix.

Overall, the trend of ultrastructural changes in SSM in both phenotypes under the hypoxic regimen of moderate severity was similar to changes in IFM. However, they were much less pronounced, which suggests that the SSM subpopulation is either better supplied with oxygen and therefore does not experience its deficit under this hypoxic regimen, or it is much more resistant to oxygen deficit, or else both factors take place.

At severe hypoxia, the average SSM area in the myocardium of HR rats sharply decreased, while the number of mitochondria, especially small ones, increased almost 2 times compared to the previous regimen and even exceeded the baseline values (Table 2 and Table 4). All this points to the activation of proliferative processes. Herewith, the characteristic feature was a chaotic arrangement of cristae. We observed a decrease in the number of intermitochondrial contacts compared to previous hypoxia regimens, which indicates the emergence of signs of energy deficit.

The other way round, in SSM of LR rats in severe hypoxia, fusion processes apparently intensified, because mitochondria got sharply enlarged, stretching in length ( Figure 6E and Figure 7E). In this case, as in the control and in previous hypoxia regimens, the membrane tightly encircled the mitochondria, which gave it a tortuous character. Herewith, cristae were packed sufficiently closely, but were arranged chaotically. It is considered that mitochondrial elongation correlates with an increased activity of OXPHOS and elevated ATP production [43,44,45]. Chains of several fused mitochondria with constrictions were also preserved ( Figure 6E and Figure 7E). However, unlike in the previous regimen, they were swollen, cristae in them were partially lysed and were arranged in a chaotic way.

Thus, the SSM subpopulation forms a clearcut response to severe hypoxic exposure, fundamentally different in HR and LR rats. In both cases, however, there were no ultrastructural changes characteristic of pathologies. On the contrary, the used mechanisms determining the ultrastructural pattern of SSM (fission or fusion) were aimed at maintaining their specific energy-dependent functions necessary for operation of the myocardium under these conditions. The absence of significant ultrastructural changes in SSM as compared with other mitochondrial subpopulations is, probably, due to its increased supply with oxygen.

c. Effect of hypoxia on the ultrastructure of PNM in the myocardium of LR and HR rats

According to the literature data, severe hypoxia causes substantial perinuclear mitochondrial clustering. Exposure to hypoxia triggers a retrograde mitochondrial movement that requires microtubules and the microtubule motor protein dynein, and results in the perinuclear clustering of mitochondria.

In our experiments, mitochondrial perinuclear localization depended on the severity of hypoxic exposure and the phenotype of animals. The reaction of PNM to hypoxia was fundamentally different in HR and LR rats.

PNM within the range of FiO_2_ = 14–10% were characterized by a decrease in both their total number and the number of small organelles (Table 5). Because of this, their ratio in this range of O_2_ concentrations did not change: small mitochondria were 55–58% of the total.

In contrast, the amount of PNM in LR rats within the range of FiO_2_ = 14–10% did not significantly change, whereas the number of small organelles increased. As a result, at FiO_2_ = 10%, the total number of PNM, as well as the number of small organelles in the subpopulations of LR and HR rats, were leveled (Table 5).

In severe hypoxia (FiO_2_ = 8%), the number of small PNM in HR animals continued to sharply decline (down to 26%). Their total number, however, increased (by 30%), which suggests an enhanced retrograde mitochondrial movement to the perinuclear region under these conditions. The average area of the PNM of HR rats reduced twofold during this period.

The average area of the PNM subpopulation of LR rats also decreased twice in severe hypoxia. Thus, the difference in size between HR and LR rats, characteristic of normoxia, remained. However, in LR, unlike HR animals, the total number of PNM and the number of small organelles increased sharply (2.3- and 3-fold, respectively). Owing to this, as in the previous hypoxic regimen, the total number of PNM and the number of small organelles in the LR and HR subpopulations were of the same order (Table 5).

Hypoxic severity-dependent differences between LR and HR subpopulations also concerned the PNM ultrastructure.

**Figure 8:**
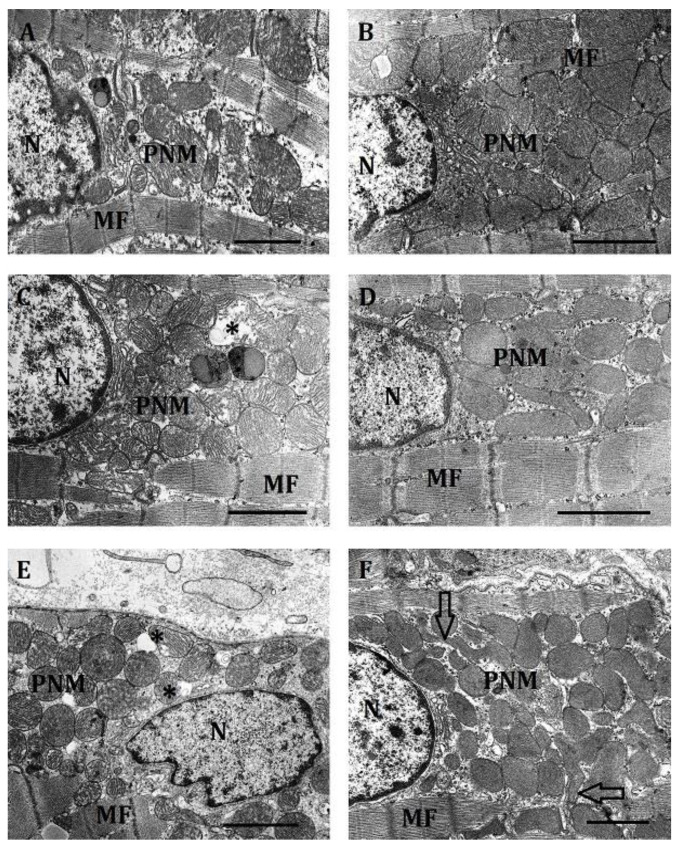
The ultrastructure of a subsarcolemmal region of a cardiomyocyte in rats with low (**A**,**C**,**E**) and high (**B**,**D**,**F**) resistance to oxygen deficit in mild (FiO_2_ = 14%, (**A**,**B**)), moderate (FiO_2_ = 10%, (**C**,**D**)) and severe (FiO_2_ = 8%, (**E**,**F**)) hypoxia. Tissue samples were taken 30 min after the start of the respective hypoxic training. SSM, subsarcolemmal mitochondria; MF, myofibrils. Arrow—nanotunnels; asterics—lysed mitochondria. Scale bar, 0.5 μm.

Under the action of mild hypoxia (FiO_2_ = 14%) in HR rat myocardium, the clustering of perinuclear mitochondria characteristic of normoxia slightly increased. However, the shape of the organelles became more polymorphic. Along with contacts of the “kissing junctions” type, nanotunnels occurred (Figure 8). Mitochondria became looser. Lumens emerged between cristae in many mitochondria.

At moderate hypoxia (FiO_2_ = 10%), because the number of PNM and the number of small mitochondria in HR rats continued to decrease, the density of organelles in the perinuclear region and their clustering decreased. The number of lumens between mitochondria increased. The shape of organelles also changed. Elongated interlocked mitochondria appeared. Their matrix, however, was sufficiently dense (Figure 8).

In severe hypoxia (FiO_2_ = 8%), clustering of PNM in HR rats was minimal. The subpopulation was a cluster of small, polymorphic, or else weakly contacting, or nanotunnel-bound organelles near the nucleus. There was a large number of cytoplasmic lumens. However, the organelle matrix was quite dense. The number of lysed mitochondria was minimal (Figure 8).

The dynamics of PNM ultrastructure in LR rats under different hypoxic regimens also differed fundamentally.

At the action of mild hypoxia, the small size of the perinuclear region characteristic of normoxia, and a small number of PNM localized there, far from the nucleus, were preserved in LR rats. The contacts between the organelles were weakened. There was a large number of lumens between mitochondria. The density of the mitochondrial matrix was reduced. The cristae were poorly ordered, had enlarged intracristal spaces and were clearly visible. In some mitochondria, lysis was observed.

At moderate hypoxia, however, the perinuclear region in cardiomyocytes of LR rats increased, as did the number of PNM. Their area near the nucleus also increased. Mitochondria in this case had a predominantly rounded shape. Contacts between them were loose, their clustering was not pronounced. Nevertheless, cytoplasmic lumens decreased dramatically. The matrix was clarified, cristae were clearly visible and were sufficiently ordered. At the same time, at moderate hypoxia, mitochondria with signs of lysis occurred.

In severe hypoxia, the overwhelming number of PNM in LR rats had a round shape, a dense matrix. Cristae were tightly packed. Mitochondria were sufficiently densely arranged relative to one another. There was a tendency towards organelle clustering. Signs of lysis were practically absent. The main feature of PNM of LR rats was their accumulation around the nucleus (Figure 8E). The nucleus itself could lose its oval shape. There were numerous invaginations on its surface.

Thus, PNM of HR and LR rats reacted clearly and differentially to changes in the O_2_ content within a wide range of FiO_2_. Changes of their ultrastructure in severe hypoxia were, however, aimed in this case at the formation of a smaller, rather diffusely arranged population of organelles with signs of high metabolic activity. This dynamics at a simultaneous decrease of signs of lysis suggests a change in the functional orientation of this subpopulation in severe hypoxia towards enhanced transcriptional activity. It was shown above that it was exactly during this period that the induction of HIF-1α and GPR91 was sharply activated.

## 3. Discussion

The heart is a highly oxygenated organ, in whose cardiomyocytes mitochondria occupy more than 30% of the volume. Under normal physiological conditions, they synthesize 50–70% of the ATP in the process of β-oxidation of fatty acids [46]. Nevertheless, the data we obtained indicate that in the myocardium a single hypoxic exposure in a wide range of oxygen concentrations (FiO_2_ = 14–8%) leads to significant activation of succinate oxidation after 15–30 min (an increase in SDHA expression, which reflects the activation of electron transport at this site). This activation is short-term and, irrespective of the duration of hypoxic exposure, after an hour it is replaced by its decrease.

At the same time, however, there is a long-lasting increase in the activity of the electron transport function of the cytochrome site of the respiratory chain and ATP synthase (an increase in the expression of cyt b, COX and ATP5A subunits), and, consequently, the activity of MC III, MC IV, MC V. Thus, the activation of succinate oxidation in the initial period of hypoxia apparently contributes to enhancing the function of the myocardial energy apparatus. According to the literature, the shortage of succinate leads to a reversible loss of membrane potential, which can be quickly restored by the addition of succinate [47]. Earlier, increased activity of respiratory enzymes and coenzymes was detected in response to staying at high altitude or in experiments simulating this condition [48,49].

Activation of succinate oxidation during hypoxia is considered as an evolutionarily formed regulatory-compensatory mechanism, which is realized in conditions of oxygen deficiency in most tissues [8,10,11,17,18,30,33,50,51,52,53,54]. According to modern concepts, succinate accumulation is the earliest marker of hypoxia [55,56,57,58,59,60,61]. Our results confirm these conclusions. They also indicate that in conditions of hypoxia, the myocardium uses this succinate-dependent mechanism in the formation of urgent adaptation.

The high efficiency of succinate oxidation was shown by Chance [62]. Under hypoxic conditions, succinate has advantages over the oxidation of NAD-dependent substrates of the tricarboxylic acid cycle (TCA), since it is able to oxidize in tissues with a lack of O_2_. Despite the fact that only two phosphorylation points are preserved, significantly higher reaction rates ensure high energy efficiency of the process as a whole.

It is known that to maintain a sufficient level of ATP, the myocardium can use any available substrates, and first of all, glucose, which leads to an increase in the content of pyruvate, activation of TCA and synthesis of the succinate [3,60,63,64,65,66]. It is believed that the combination of glucose/fatty acid substrates in hypoxia provides the best functioning of the heart [67]. Such lability in the choice of energy substrate should be considered as a manifestation of urgent adaptation of the myocardium to the changed conditions of functioning.

It is important to note that the activation period of succinate oxidation in the myocardium is short-term and after an hour its decrease begins even if the hypoxic exposure continues. It is known that prolonged accumulation of succinate may cause a disturbance of physiological regulatory mechanisms of adaptation and lead to undesirable side effects, up to the development of pathologies often described in the literature [26,28,34,54,59,60,61,62,68,69].

The data obtained in our study also indicate the existence in the myocardium, as in the brain cortex [8,9,10,70], of reciprocal relations between the activity of NAD- and FAD-dependent oxidation pathways, having regulatory significance. During the intensification of SDHA expression, either there were no significant changes in the level of NDUFV2 or its decrease was observed. However, at the peak of SDHA expression or during its decline, there was the increase in NDUFV2 expression, reached maximum in the post-hypoxic period. For methodological reasons, measuring the content of respiratory chain enzymes in our work in the earlier period (5–10 min) was hindered. However, presumably the activation of NAD-dependent oxidation (MC I) appears already in the first minutes of hypoxic exposure that, [8,71,72] can be then replaced by activation of FAD-dependent oxidation (MC II). In some studies, a decrease or even suppression of the activity of MC I in hypoxia/ischemia is assessed as a sign of mitochondrial dysfunction [53,73]. However, as follows from our experiments, this process appears against the background of an increase in ATP synthase activity and ended with the restoration of MC I function at the end of hypoxic exposure or immediately after it. Consequently, in the myocardium mitochondria, this process reflects the change of metabolic flows, which ensures the use of the most energetically efficient substrates and due to this allows to stabilize the functioning of the heart for a certain period of time [74,75].

Thus, in response to a single hypoxic exposure, mechanisms are activated in the myocardium mitochondria that allow for subtle differentiation of urgent changes in the O_2_ content in the environment, control the flow of energy substrates, affecting the interaction of MC I and MC II and increasing the efficiency of OXPHOS.

There are several mechanisms that might be involved in the modulation of substrate flows and activation of succinate oxidation in the myocardium at the early stage of acute hypoxia. For example, it has been proved that there is a metabolic relationship of oxidative processes in mitochondria with the adrenergic and cholinergic systems [76,77,78,79,80]. Stimulation of physiological functions by adrenaline involves selective activation of succinate dehydrogenase (SDH), and succinate, in turn, is a signaling molecule that stimulates the release of adrenaline and noreadrenaline. A similar relationship was found between α-ketoglutarate and acetylcholine [76,77].

The effects of sympathetic activation, contributing to the improvement of the function of the mitochondria of the heart, muscles and other tissues, depend on the classical Gas-cAMP-PKA pathway, stimulating the release of Ca^2+^ [81,82,83,84,85]. Besides, mitochondria have extensive interactive networks with many organelles inside the cell, primarily with the endoplasmic reticulum. These interactions result in Ca^2+^ signaling [71]. There are data that succinate affects the mitochondrial calcium signal [86]. Moreover, succinate is one of the metabolites which increase the expression of transcriptional regulators of glucose utilization and lipid metabolism genes [28,87]. At the systemic level, succinate increases cardiac output in ischemia and hypoxia [33,34].

The AMP/ATP ratio is the main regulator of the OXPHOS in a certain region of reduced O_2_ values, while the ATP/ADP ratio looses this role under these conditions. [88,89]. An important regulatory protein of energy metabolism in this case is AMPK (AMP-activated protein kinase). It regulates energy metabolism by phosphorylating a number of key metabolic enzymes, modulating their activity and increasing energy production (glucose transport, fatty acid oxidation). Any decrease in O_2_ content, for example, during an ischemic episode, will inevitably evoke an energy imbalance, an increase in the AMP/ATP ratio and, thus, activation of AMPK. It is known that AMPK is an urgent switch of glucose and lipid metabolism in various organs, especially in the heart, skeletal muscles and liver [89,90,91,92,93,94,95,96,97,98]. Thus, urgent adaptation in heart cells can be achieved through metabolic signaling via phosphotransfer networks. These processes can increase the rate of ATP production without significant changes in thermodynamic forces.

According to modern views, the mitochondrial respiratory chain is involved in various intracellular signaling programs and performs the role of a signaling transformative metabolic system that activates the functional response and the physiological reactions of the organism to a variety of impacts, and above all, to hypoxia [99,100,101,102,103,104,105,106,107,108,109,110,111]. As noted above, succinate-dependent signaling pathways induced by adaptive factors GPR91, VEGF, and HIF1-α play a special role in this process [24,25,26,27,30,31,35,36,37].

From the data we obtained, it follows that only succinate-dependent induction of HIF-1α was observed in the myocardium in response to single hypoxic exposure of mild and moderate severity. HIF-1α enhanced glucose metabolism by inducing the expression of GLUT1 and several glycolytic genes [112,113]. In addition, it induced the expression of non-metabolic targets, including genes that regulate erythropoiesis (e.g., erythropoietin) and angiogenesis (e.g., VEGF) to increase the body’s ability to deliver oxygen to cells in response to chronic hypoxia. Under the conditions of our experiment, however, no reliable induction of VEGF was observed. No succinate-dependent expression of GPR91 has been detected, either. Consequently, at mild and moderate hypoxia, the function of the heart in the first 30–45 min of hypoxic exposure was maintained by basic physiological reserves. After that, a short-term succinate-dependent induction of HIF-1α was observed. Consequently, at mild and moderate hypoxia, the function of the heart in the first 30–45 min of hypoxic exposure was maintained by basic physiological reserves.

However, in severe hypoxia, urgent induction of not only HIF-1a, but also GPR91 developed simultaneously. In both cases, their expression was significantly ahead of SDHA expression, i.e., it was succinate-independent. In conditions of severe O_2_ deficiency, it is necessary to mobilize a wider range of adaptive signaling systems that could ensure the preservation of the myocardial contractile function. Thus, the nature of the induction of signaling systems in the myocardium reflects the severity of hypoxic exposure.

In severe hypoxia, urgent induction of not only HIF-1α but also GPR91 developed simultaneously. In both cases, their expression was significantly ahead of SDHA expression, i.e., was succinate-independent. Consequently, under conditions of severe O_2_ deficit, it was necessary to mobilize a wider range of adaptation signaling systems that could ensure the preservation of myocardial contractile function. Thus, the character of the induction of signaling systems in the myocardium reflects the severity of hypoxic exposure.

The ultrastructure of mitochondria and their structural/morphological dynamism (mitochondrial dynamics) are closely related to the energy function of mitochondria and their metabolism. We have demonstrated these interrelated relationships under hypoxic conditions earlier on cerebral cortex mitochondria [70]. In the myocardium, unlike most other tissues, there are three subpopulations of mitochondria (IFM, SSM and PNM). Their roles in the vital activity of the heart have not yet been sufficiently studied. Nevertheless, it is obvious that they differ in intramitochondrial localization, morphology, functions and different responses to external effects [4,19,39,114,115].

It has been shown, for example, that IMF, the most static and numerically large subpopulation [20], are characterized by high rates of ATP synthesis, respiration, substrate oxidation, succinate dehydrogenase and citrate synthase activities [19,116,117,118]. It has been also shown that the main function of IMF is to produce ATP for cardiomyocyte contraction and for Ca^2+^ signal transfer from the sarcoplasmic reticulum to mitochondria [4,6,7,116,119,120].

SSM, quite the contrary, is the smallest group located immediately under the cardiomyocyte plasma membrane or in areas where cardiomyocytes come close to blood capillaries. There is evidence that SSM are involved in providing the energy necessary for active transport of oxygen from erythrocytes to cardiomyocytes, for phosphorylation of some sarcolemmal proteins, as well as for transport of ions and metabolites across the sarcolemma.

The low ΔΨ characteristic of SSM allows these mitochondria to maintain a low intracellular oxygen concentration and to restrict the formation of reactive oxygen species. For this reason, SSM can play a crucial role in antioxidant protection by regulating both the diffusion of oxygen and its concentration inside the cell.

Unlike IMF and SSM, PNM, grouped around the nucleus and not associated with myofibrils, are relatively motile, participate in fission/fusion dynamics, play a central role in mitochondrial genesis and transcription processes, and generate most of the ATP required for nuclear processes [121].

The present work was the first to reveal the functional and phenotypic heterogeneity of these myocardial mitochondrial subpopulations and their ability to finely differentiate the O_2_ content in the inhaled air.

HR as compared with LR rats were observed to have a tighter packing of cristae in IFM and SSM, a darker matrix, a larger total number and the number of small IFM and PNM, and a significantly larger area and diameter of PNM. An increased number of mitochondria is known to provide their resistance to oxygen deficit [39], so a reduced number of mitochondria, especially of small diameter, and reduced packing of cristae, are indicators of reduced mitochondrial functional activity [122,123]. Besides, the increased content of PNM in the myocardium of HR rats is indicative of a greater ability of these animals to synthesize proteins that regulate the adaptation to hypoxia [123].

Cardiomyocytes of two investigated phenotypes of animals at normoxia were also found to have different configurations of the sarcolemmal membrane. SSM organelles in LR rats are, as a rule, located in the invaginations of the sarcolemmal membrane, giving it a tortuous shape not observed in HR animals. This increased the contact area of the SSM with the sarcolemma, which allows them to capture more oxygen from nearby vessels for better energy supply. This membrane configuration is preserved in LR rats during hypoxia.

Phenotypic differences are especially pronounced in the IFM subpopulation. Under conditions of mild and moderate oxygen deficit, we observed an increasing modification of the IFM ultrastructure, indicating an increase in the degree of energization, pronounced the most at FiO_2_ = 10%. The dynamics of IFM differed in LR and HR rats, but in both cases it was aimed at preserving the myocardium performance. These changes correlated with those in the activity of enzymes of the respiratory-chain cytochrome site and ATP synthase. In contrast, under severe hypoxic conditions (FiO_2_ = 8%), the ultrastructural pattern of IFM was characterized by obvious signs of deenergization, though no quantitative changes were observed in the enzymes of the respiratory-chain cytochrome site. Therefore, changes in the ultrastructural pattern of IFM during hypoxia are the most sensitive markers of dysfunction of the myocardial energy apparatus.

The trend of ultrastructural changes in SSM of both phenotypes under mild and moderate hypoxic regimens was similar to that in IFM, but significantly less pronounced, which can be due to a better supply of oxygen to SSM. However, the response of SSM to severe hypoxic exposure was fundamentally different from the dynamics of IFM. Only minor signs of energy deficit emerged in SSM of HR animals, and proliferative processes were intensified. In SSM of LR rats, the other way round, fusion processes appeared to be intensified, mitochondria were sharply enlarged, stretching in length. At the same time, cristae were packed rather tightly but were arranged chaotically. Mitochondrial elongation is thought to correlate with increased OXPHOS activity and ATP production [43,44,45]. At the same time, both phenotypes had no ultrastructural changes characteristic of pathologies. Thus, even under severe hypoxic conditions, SSM retained the ability to synthesize the energy required to realize their specific functions related to ion transport and maintenance of oxygen homeostasis in the cardiomyocyte.

As for PNM, their perinuclear mitochondrial localization also depended on the severity of hypoxic exposure but was fundamentally different in the two animal phenotypes. In HR rats, we observed a gradual decrease in clustering of organelles, their dissociation, an increase in the lumens between them, a polymorphism, and a matrix density decrease. Nanotunnels emerged. The number of lysed mitochondria was minimal.

In LR rats under these conditions, there was either no clustering or it was insignificant, the density of the matrix was low, cristae were disordered. At the same time, mitochondria with partially lysed cristae did occur.

All these differences suggest that, under mild and moderate hypoxia, nuclear–mitochondrial interactions were more pronounced in PNM of HR rats, whereas processes of mitochondrial population renewal prevailed in PNM of LR animals.

It should be emphasized that severe hypoxia induced mitochondrial fragmentation in both HR and LR animals. In both cases, PNM were represented by a cluster of small, polymorphic or weakly contacting, or else nanotunnel-bound (in HR) organelles localized near the nucleus. A large number of cytoplasmic lumens was observed. The organelle matrix was quite dense (to a greater extent in HR than LR rats). The number of lysed mitochondria was minimal. These dynamics, at a simultaneous decrease in the signs of lysis, suggests an increase in transcriptional activity at severe hypoxia in the PNM of both phenotypes.

According to the literature data, mitochondria grouped near the nucleus contribute to the accumulation of reactive oxygen species in the nucleus. This, in turn, activates the generation of ATP necessary for nuclear processes, for transcription including [121]. According to the literature data, mitochondria grouped near the nucleus contribute to the accumulation of reactive oxygen species in the nucleus. This, in turn, activates the generation of ATP necessary for nuclear processes, for transcription including [121].

A hypoxic enhancement of transcriptional activity can be the reason for a sharp increase in urgent succinate-independent induction of HIF-1α and GPR91 in the myocardium we observed under this hypoxic regimen

It is known that the nucleus of a normoxic cardiomyocyte is usually located in the center of the cell, i.e., far from the vessel. For this reason, the observed approach of nuclei to the vessel under severe hypoxia, more pronounced in HR rats, is probably a sign of adaptation to oxygen shortage. This movement of the nucleus and the displacement of mitochondria to the sarcolemma (vessel) has already been described in the literature and is observed both in a number of physiological conditions and in various pathologies [121,124].

The data we obtained indicate that the formation of urgent adaptation to hypoxia in the myocardium begins from the moment of the first hypoxic exposure. This is a very short period of generalized reaction to an irritant, associated not only with the mobilization of physiological mechanisms (regulatory centers of the respiratory and cardiovascular systems, oxygen transport), but also with the activation of intracellular molecular processes aimed at preserving and maintaining energy synthesis. Depending on the strength of the primary impact on the myocardium during this period, mechanisms are activated that ensure the formation of an urgent protective reaction at the cellular and systemic levels, and may be involved in the formation of delayed gene-dependent effects of long-term adaptation.

Thus, urgent adaptation of the myocardium to hypoxia includes multilevel changes in the regulatory control of key molecular components of metabolic pathways involving mitochondria, as well as dynamic changes of the ultrastructure and the subcellular localization of mitochondria themselves.

Understanding the interrelation between hypoxia signaling and mitochondria is of great theoretical and practical importance and therefore warrants continued investigation and expansion of our knowledge.

## 4. Materials and Methods

### 4.1. Evaluation of Animals’ Resistance of to Hypoxia

Experiments were performed on outbred rats with different baseline resistance to oxygen shortage. Tolerance of acute hypobaric hypoxia (HBH) was evaluated one month prior to the experiment [16,70]. The ability of rats to stay in the altitude chamber at a simulated subcritical altitude (11,000 m; time to abnormal breathing patterns, Tr resistance index) was assessed. (The critical, life-incompatible altitude for rats was 13,000–14,000 m). Tr characterizes the viability of animals under extreme hypoxic conditions and reflects the ability to fully mobilize the nonspecific protective functions responsible for survival in the sublethal period.

After registering Tr, the pressure in the chamber was normalized to the “sea level”, and the animals restored the normal posture and locomotor activity within 4–6 min. The Tr value was 1–2 min for control LR rats and more than 8 min for control HR rats. Typically, in a sample of 100 rats, 30–35% were LR to acute hypoxia (Tr < 2 min), 20–25% were HR (Tr > 6–8 min) and 40–50% were mid-resistant (Tr = 3–5 min).

### 4.2. Hypoxia Regimens

LR and HR rats were subjected to a single hypoxic exposure to three HBH regimens: (1) (FiO_2_ = 14%; HBH 523 Torr, 3000 m), mild hypoxic exposure; (2) (FiO_2_ = 10.5%, HBH 380 Torr, 5000 m), moderate hypoxic exposure; (3) (FiO_2_ = 8%, HBH 290 Torr, 7000 m), severe hypoxic exposure.

The control group was kept outside the hypobaric chamber in the same location. After the experiment, all rats were alive and resumed their normal activity without any sign of pathology.

### 4.3. Electron Microscopy of the Heart

After decapitation of rats, the isolated fragments of the heart were immediately fixed in 2.5% glutaraldehyde in 0.1 M cacodylate buffer (pH 7.4) for 2 h and then additionally fixed in 2% osmic acid prepared in the same buffer as described by Weakley [125]. Heart preparations for electron microscopy were further prepared as described earlier [18].

The total number and the number of small mitochondria (perimeter, 0.14–0.25 μm) were counted and expressed in units per plate (10 μm^2^). At least 50 negatives for one experimental group were analyzed. The number and size, perimeter and area of mitochondria were determined using the Image J program.

Morphometrical data were analyzed using the Prizm for Windows (version 5.0) software.

### 4.4. Western Blot Analysis

In connection with the objectives of the study, the following parameters were investigated using Western blot analysis: (A) the catalytic iron-containing subunits of mitochondrial myocardial complexes responsible for electron-transport function (MC-I subunit, NDUFV2 (NADH dehydrogenase [ubiquinone] flavoprotein 2); MC-II subunit, SDHA (flavochrome subunit A of succinate dehydrogenase), MC-III subunit, Cyt b (cytochrome b), MC-IV subunit, COX(cytochrome c oxidase subunit) and ATP5A (ATP synthase alpha chain); (B) the expression level of proteins-inducers of succinate-dependent signaling pathways (the succinate receptor (GPR91/SUCNR1), VEGF (vascular endothelial growth factor) and HIF-1α (transcription hypoxic factor).

To assess the effect of each hypoxic regime, 7 groups of animals were formed (both LR and HR): (1) control; (2–5) hypoxic effects (15–30–45–60 min); (6–7) after hypoxic exposure (2 h and 24 h).

After decapitation of rats, the heart was rapidly excised and washed in ice-cold normal saline. The isolated fragments of the left ventricular myocardium were separated and stored in liquid nitrogen until all the samples were collected. The frozen samples were ground in liquid nitrogen. Prior to biochemical studies, the myocardium was homogenized in liquid nitrogen. The homogenate was next lysed in a cooled buffer (50 mmol HEPES pH 7.6, 150 mmol NaCl, 2 mmol EGTA, 1% triton-X-100, 10% glycerin, 1 mmol dithiothreitol, 1 mmol Na_3_VO_4_, 1 mmol AEBSF, 60 μg/mL aprotinin, 10 μg/mL leupeptin, and 1 μg/mL pepstatin A) for 30 min.

The contents of the mitochondrial enzyme complex subunits, GPR91 and VEGF were measured in the non-nuclear fraction [126]. The content of transcription factor HIF-1α was measured in the myocardium nuclear extract [126,127].

The supernatant (the cytoplasmic extract) [128] containing the target proteins was collected after centrifugation (30 min, 14,000× *g*; 4 °C), mixed with the loading buffer (4× Laemmli Sample Buffer), incubated for 5 min at 95 °C, and stored at −80 °C. For the extraction of nuclear proteins, two lysis buffers were used: cytoplasmic and nuclear [122,123].

The protein concentration in the samples was defined spectrophotometrically by a Bradford assay. The proteins of the prepared samples were separated in 10% polyacrylamide gel and transferred into a nitrocellulose membrane via electroelution. Non-specific antibody binding was blocked by incubation in 5% defatted milk containing PBS and 0.1% Tween-20 for 1 h. The incubation was performed overnight at 4 °C in a solution of the primary monoclonal antibodies (Santa Cruz Biotechnology Inc., Santa Cruz, CA, USA, 1:500); mouse antibody against NDUFV2 (sc–515589), SDHA (sc–166909), cyt c1 (sc–514435), COX2 (sc–514489), ATP5A (sc–136178), VEGF (sc–365578), HIF-1a (sc-10790), and the first polyclonal antibody, rabbit anti-GPR91 (Abcam plc, Cambridge, UK, ab41505). Anti-actin antibodies (sc–376421) were used as controls. Proteins were detected by reaction with ECL reagents (Pierce Biotechnology, Inc., Waltham, MA, USA) on Kodak film followed by densitometry in Adobe Photoshop software. The protein content was estimated by optical density of the band reflecting the antibody binding to the protein. The result was expressed as relative densitometric units (RDU).

Statistical data analysis was performed using the Statistica 10.0 software package (Stat Soft Inc., Tulsa, OK, USA) using the Wilcoxon (Wilcoxon–Mann–Whitney) nonparametric rank U-test. Differences between the compared groups were considered statistically significant at *p* < 0.05.

## 5. Conclusions

In summary, urgent adaptation of the myocardium to various modes of single hypoxic exposure is achieved by the following: (1) Urgent remodeling of the respiratory chain (short-term activation of succinate oxidation, activation of enzymes of the cytochrome site of the respiratory chain and ATP synthase); (2) Induction of HIF-1α-dependent signaling in mild and moderate hypoxia and induction of HIF-1α- and GPR91- dependents signaling in severe hypoxia; (3) Differentiated modification of the ultrastructure of three subpopulations of myocardial mitochondria.

The most sensitive prognostic criterion of the state of the myocardial energy apparatus in various hypoxic regimes is the dynamics of IFM. Hypoxia-induced perinuclear clustering of IFM correlating with succinate-independent urgent induction of HIF-1a and GPR9 may be associated with the activation of transcription processes.

## Figures and Tables

**Table 1:** Morphometric characteristics of three subpopulations of mitochondria in the myocardium of LR and HR rats under normoxic conditions.

Type of Mitochondria	LR, Units/10 µm^2^		HR, Units/10 µm^2^	
	Avg. Number of Small mt (perimeter ≤ 0.25 µm)	Avg. Number of Medium and Large mt	Avg. Number of Small mt (Perimeter ≤ 0.25 µm)	Avg. Number of Medium and Large mt
IFM	1.41 ± 0.97, 12%	9.89 ± 0.211, 88%	2.48 ± 1.47, 15%	13.56 ± 2.84, 85%
SSM	1.92 ± 0.86, 18%	9.02 ± 0.189, 82%	1.82 ± 0.69, 16%	9.84 ± 1.79, 84%
PNM	6.60 ± 1.67, 37%	11.40 ± 2.85, 63%	15.92 ± 3.96 *, 55%	12.88 ± 2.96, 45%

*m* ± SD; * the differences between the groups are significant, *p* < 0.05; mt, mitochondria.

**Table 2:** Stereometric characteristics of IFM, SSM and PNM in the myocardium of HR and LR rats after a single exposure to different HBH regimens.

FiO_2_	Average Area of IFM, µm^2^		Average Area of SSM, µm^2^		Average Area of PNM, µm^2^	
	LR Rats	HR Rats	LR Rats	HR Rats	LR Rats	HR Rats
Control	0.673 ± 0.430	0.685 ± 0.460	0.485 ± 0.291	0.535 ± 0.260	0.453 ± 0.046	2.556 ± 0.154
14%	0.508 ± 0.360 **	0.543 ± 0.394 *	0.496 ± 0.252	0.443 ± 0.354	0.319 ± 0.030	2.143 ± 0.192
10%	0.745 ± 0.586	0.844 ± 0.740 **	0.564 ± 0.230	0.644 ± 0.340	0.586 ± 0.084	2.705 ± 0.222
8%	0.512 ± 0.353 **	0.660 ± 0.527	0.532 ± 0.327	0.460 ± 0.227	0.241 ± 0.019 *	1.827 ± 0.081 *

*m* ± SD; * *p* < 0.05, ** *p* < 0.001.

**Table 3:** Morphometric characteristics of IFM in the myocardium of HR and LR rats after a single exposure to different HBH regimens.

FiO_2_	LR, Units/10 µm^2^		HR, Units/10 µm^2^	
	Total Number of IFM	Average Number of Small IFM	Total Number of IFM	Average Number of Small IFM
Control	11.30 ± 2.85 (*n* = 27, mt = 305)	1.41 ± 0.97	16.04 ± 4.65 (*n* = 23, mt = 369)	2.48 ± 1.47
14%	15.56 ± 4.08 * (*n* = 16, mt = 249)	4.44 ± 1.63 *	13.89 ± 3.86 (*n* = 9, mt = 125)	3.56 ± 2.60
10%	11.67 ± 4.20 (*n* = 15, mt = 175)	2.47 ± 1.25 *	8.96 ± 3.41 * (*n* = 27, mt = 242)	1.44 ± 1.42
8%	17.00 ± 3.79 * (*n* = 16, mt = 272)	3.88 ± 2.19 *	13.93 ± 5.43 (*n* = 14, mt = 195)	3.50 ± 2.14

*m* ± SD; * *p* < 0.05; mt, mitochondria.

**Table 4:** Morphometric characteristics of SSM in the myocardium of HR and LR rats after a single exposure to different HBH regimens.

FiO_2_	LR, Units/10 µm^2^		HR, Units/10 µm^2^	
	Total Number of SSM	Average Number of Small SSM	Total Number of SSM	Average Number of Small SSM
Control	10.94 ± 2.25 (*n =* 17, mt = 186)	1.92 ± 0.86	11.65 ± 3.14 (*n =* 23, mt = 199)	1.84 ± 0.69
14%	8.58 ± 2.75 (*n =* 12, mt = 103)	1.56 ± 0.90	7.41 ± 1.86 (*n =* 17, mt = 126)	0.84 ± 0.63
10%	6.86 ± 3.86 (*n =* 14, mt = 96)	0.54 ± 0.62	6.89 ± 2.67 (*n =* 19, mt = 131)	1.47 ± 1.25
8%	8.78 ± 2.51 (*n =* 9, mt = 79)	1.50 ± 0.84	10.13 ± 3.26 (*n =* 8, mt = 81)	1.88 ± 0.91

*m* ± SD; mt, mitochondria.

**Table 5:** Morphometric characteristics of PNM in the myocardium of HR and LR rats after a single exposure to different HBH regimens.

FiO_2_	LR, Units/10 µm^2^		HR, Units/10 µm^2^	
	Total Number of PNM	Average Number of Small PNM	Total Number of PNM	Average Number of Small PNM
Control	18.00 ± 3.31 (*n* = 18, mt = 324)	6.60 ± 1.67	28.80 ± 5.82 ^$^ (*n* = 15, mt = 432)	15.92 ± 3.96 ^$^
14%	16.10 + 2.80 (*n* = 20, mt = 322)	7.24 ± 2.56	23.46 ± 3.67 (*n* = 13, MX = 305)	13.66 ± 2.57 ^$^
10%	18.79 + 3.04 (*n* = 14, mt = 263)	12.16 ± 3.85	21.50 ± 5.79 (*n* = 16, MX = 344)	11.84 ± 4.18
8%	41.87 ± 6.00 * (*n* = 15, mt = 628)	20.50 ± 5.72 *	37.80 ± 3.08 * (*n* = 15, MX = 567)	9.86 ± 2.13

*m* ± SD; mt, mitochondria. * the differences between the control and hypoxia in the group are significant, *p* < 0.05. ^$^ the differences between groups LR and HR, *p* < 0.05.

## Data Availability

The data presented in this study are available on request from the corresponding authors.

## Abbreviations

ADP	adenosine diphosphate
AMP	adenosine monophosphate
ATP5A	subunit of MC V (ATP synthase F1 subunit alpha)
AMPK	AMP-activated protein kinase
ATP	adenosine triphosphate
Cyt b	subunit of MCIII (cytochrome b)
COX2	subunit of MC IV (cytochrome *c* oxidase subunit 1)
FiO2	oxygen content in inhaled air (%)
GPR91	Succinate G protein-coupled receptor
HBH	acute hypobaric hypoxia
HIF-1α	hypoxia-inducible factor 1 alpha subunit
HR	high resistant rats
IFM	interfibrillar mitochondria
LR	low resistant rats
MC	mitochondrial complex
NDUFV2	a subunit of complex I (MC I, NADH dehydrogenase [ubiquinone] flavoprotein)
OXPHOS	oxidative phosphorylation
PNM	perinuclear mitochondria
SDHA	subunit of succinate dehydrogenase complex (MC II)
SSM	subsarcolemmal mitochondria
VEGF	vascular endothelial growth factor
